# Initial Analysis of Structural Variation Detections in Cattle Using Long-Read Sequencing Methods

**DOI:** 10.3390/genes13050828

**Published:** 2022-05-06

**Authors:** Yahui Gao, Li Ma, George E. Liu

**Affiliations:** 1 Animal Genomics and Improvement Laboratory, Beltsville Agricultural Research Center, Agricultural Research Service, U.S. Department of Agriculture, Beltsville, MD 20705, USA; gyhalvin@gmail.com; 2 Department of Animal and Avian Sciences, University of Maryland, College Park, MD 20742, USA; lima@umd.edu

**Keywords:** cattle, structural variation, long-read sequencing

## Abstract

Structural variations (SVs), as a great source of genetic variation, are widely distributed in the genome. SVs involve longer genomic sequences and potentially have stronger effects than SNPs, but they are not well captured by short-read sequencing owing to their size and relevance to repeats. Improved characterization of SVs can provide more advanced insight into complex traits. With the availability of long-read sequencing, it has become feasible to uncover the full range of SVs. Here, we sequenced one cattle individual using 10× Genomics (10 × G) linked read, Pacific Biosciences (PacBio) continuous long reads (CLR) and circular consensus sequencing (CCS), as well as Oxford Nanopore Technologies (ONT) PromethION. We evaluated the ability of various methods for SV detection. We identified 21,164 SVs, which amount to 186 Mb covering 7.07% of the whole genome. The number of SVs inferred from long-read-based inferences was greater than that from short reads. The PacBio CLR identified the most of large SVs and covered the most genomes. SVs called with PacBio CCS and ONT data showed high uniformity. The one with the most overlap with the results obtained by short-read data was PB CCS. Together, we found that long reads outperformed short reads in terms of SV detections.

## 1. Introduction

**Figure 1:**
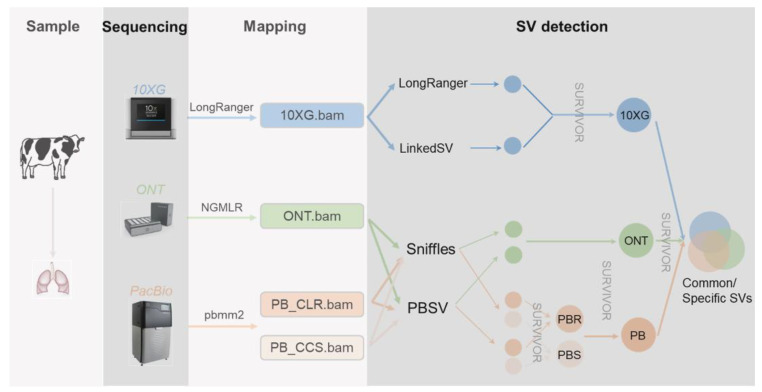
Sample collecting, sequencing, and mapping pipeline.

Unraveling the genetic underpinnings of phenotypic variation relies on a comprehensive knowledge of all forms of genetic variation. The exploitation of genetic variation has mainly focused on single-nucleotide polymorphisms (SNPs) and small insertions or deletions (indels, <50 bp), with a minor emphasis on larger variations such as copy number variations (CNV) and other structural variations (SV). SVs are most commonly defined as genomic changes of at least 50 bp in size, and they are difficult to detect precisely. Although there exist fewer SVs in the genome relative to SNPs and indels, SVs can impact more base pairs, thus being more likely to affect the phenotype [1,2]. While short-read sequencing technologies can detect SVs, they have various weaknesses. Since short reads (<1 kb) are typically smaller than or similar in size to SVs, a wide collection of indirect methods has been developed to infer SVs, including the use of split reads, read pairs, read depths, and local de novo assembly. On the other hand, linked reads provide long-range (100+ kb) information to short reads, bringing the reads into phase for haplotype-specific deletion detection, large SV detection [3,4,5], and diploid de novo assembly [6]. Long reads (>> 1 kb) spanning more SVs allow further SV detection, with mapped reads [7,8], local assembly after phasing long reads [9], and global de novo assembly [10,11]. Currently, Pacific Biosciences (PacBio) and Oxford Nanopore Technologies (ONT) are the most commonly employed technologies to produce long reads. Single-molecule real-time (SMRT) sequencing, developed by PacBio, can yield reads of tens of kilobases using either continuous long reads (CLR) or circular consensus sequencing (CCS) mode, which achieves high-quality genome assembly. ONT enables direct and real-time sequencing of long DNA or RNA by analyzing the current interference caused by the molecules as they pass through the protein nanopore. To date, these sequencing methods have enabled the improved genome assemblies for many species, including humans [12,13], cattle [14,15,16], buffalo [17], pigs [18,19], sheep [20], and goats [21]. To study the effects of these methods on SV detection in humans, Aganezov et al. [22] performed whole-genome sequencing of the SKBR3 breast cancer cell line and patient-derived tumor and normal organoids from two breast cancer patients using Illumina/10× Genomics, PacBio, and ONT sequencing. They inferred SVs and large-scale CNVs and showed that long-read sequencing enables more accurate and sensitive SV detection. In dairy cattle, Couldrey et al. [15] detected CNVs using PacBio long-read and Illumina sequencing. In this study (Figure 1), we sequenced one cattle individual using cutting-edge technologies, i.e., 10× Genomics (10 × G), PromethION (ONT), PacBio continuous long reads (PB CLR), and PacBio circular consensus sequencing (PB CCS). We then evaluated various methods using these data from the same lung DNA sample for their abilities for the SV detection.

## 2. Materials and Methods

Under the approval of the US Department of Agriculture, Agricultural Research Service, Beltsville Agricultural Research Center’s Institutional Animal Care and Use Committee (Protocol 16-016), lung tissue was collected and then snap-frozen in liquid N2 immediately after excision and kept at −80 °C until use. The high-molecular-weight (HMW) DNA for lung tissue was extracted according to the MagAttract HMW DNA Kit (Cat. No. 67563, QIAGEN, Valencia, CA, USA). The quality of DNA samples was evaluated using the 2100 Bioanalyzer and the 4200 TapeStation (both from Agilent Technologies, Santa Clara, CA, USA), including degradation, potential RNA contamination, purity (OD260/OD280), and concentration using spectrophotometers of Qubit (Thermo Fisher Scientific, Waltham, MA, USA) and NanoDrop (NanoDrop Technologies, Rockland, DE, USA) to meet the demands for library construction.

The HMW DNA was sequenced using the Linked-Reads method developed by 10× Genomics [4], and standard protocols were followed in this study. By using microfluidics to segment and barcode HMW DNA, 10× Genomics can provide long-range information for short reads of the genome. We then aligned 10 × G short reads with LongRanger [23] v2.1.6 and used LongRanger [23] v 2.1.6 and LinkedSV [24] with the recommended settings to call SVs, respectively. DNA was prepared using standard ONT methods and sequenced on a PromethION device. We aligned ONT long reads with NGMLR [8] v0.2.7 and run Sniffles [8] v1.0.11 and PBSV v2.2.0 (https://github.com/PacificBiosciences/pbsv, accessed on 3 May 2022) with default settings for SV inference. PacBio sequencing was carried out on a Pacific Biosciences Sequel II platform using two modes, i.e., continuous long reads (CLR) and circular consensus sequencing (CCS). We aligned the long reads with pbmm2 v1.3.0 (https://github.com/PacificBiosciences/pbmm2, accessed on 3 May 2022) and run Sniffles v1.0.11 [8] and PBSV v2.2.0 (https://github.com/PacificBiosciences/pbsv, accessed on 3 May 2022) with default settings for SV inference. We mapped all reads against the latest cattle genome reference ARS-UCD1.2 [25] and performed follow-up SV detection. We computed the alignment coverage by SAMtools [26] v1.9 depth command. For each sequencing technology, we merged the SVs generated by different callers with the SURVIVOR [27] v1.0.7 into a 10 × G, ONT, and PacBio technology-specific SV call sets. We then ran the SURVIVOR merge module with a maximum allowed distance of 200 bp and minimum SV size set to 30 bp regardless of SV types, as different methods may assign different types.

## 3. Results

### 3.1. SV Inference

**Figure 2:**
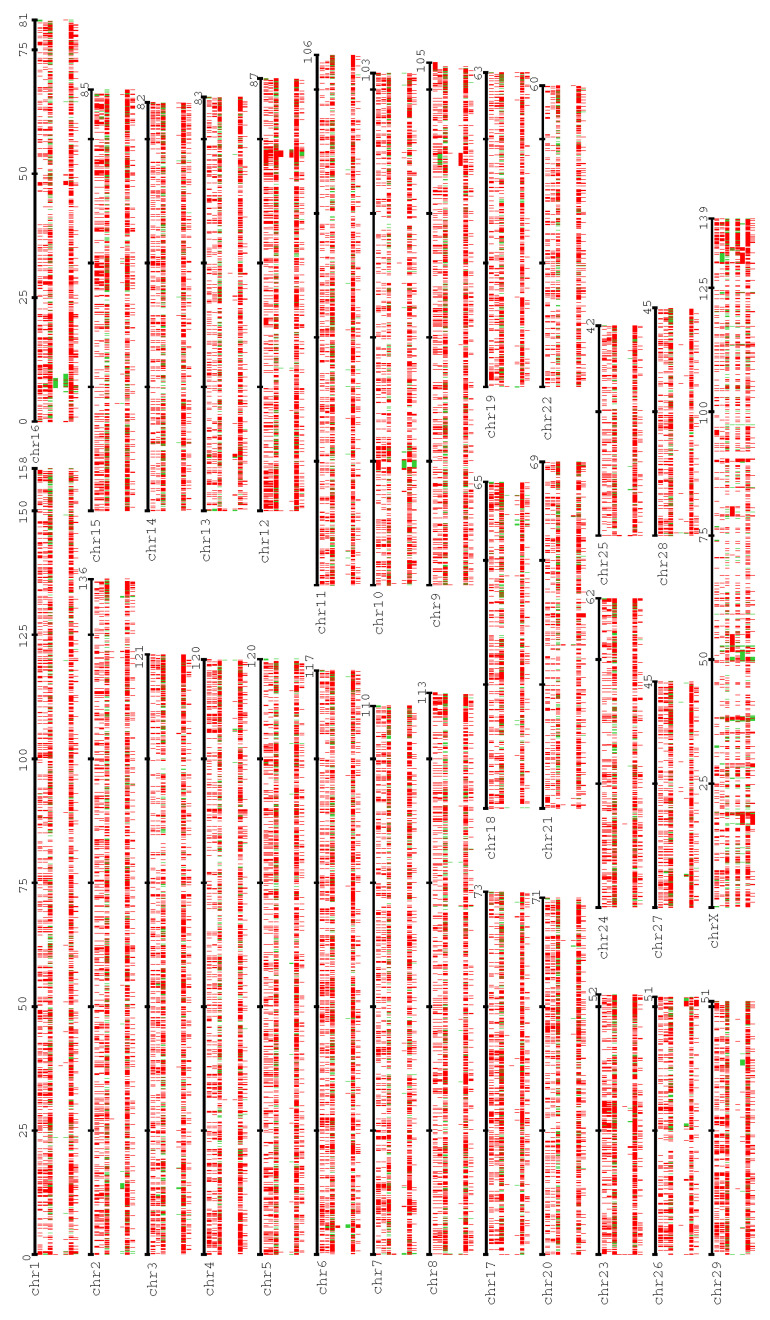
Individualized cattle SV map. The tracks under every black bar represent the SVs for 10 × G_LongRanger, 10 × G_LinkedSV, CCS_PBSV, CCS_Sniffles, CLR_PBSV, CLR_Sniffles, ONT_PBSV and ONT_Sniffles (in order from top to bottom). Red means deletion, and green means duplication.

A total of 1,577,259,728 (Table 1) short reads were generated through 10× Genomics, representing 55× coverage of the genome. The LongRanger alignment resulted in 97.14% (Table 1) of the reads mapping to the ARS-UCD1.2 cattle genome reference [25]. There was a total of 8315 and 6453 putative SVs identified by LongRanger and LinkedSV, respectively (Table 2). The SVs identified by LongRanger ranged in size from 49 bp to 1.59 Mb with an average size of 4481 bp (Table S1). For LinkedSV, the size ranged from 39 bp to 2.39 Mb, and the average size was 3180 bp (Table S1). The distribution of SVs across the genome was shown in Figure 2. After merging using SURVIVOR, the total quantity of SVs was 10,439 (114 duplications and 10,325 deletions) (Table 2), covering 53 Mb of the whole genome (Table S1).

Oxford nanopore sequencing generated 1,618,623 sequences representing approximately 11× coverage of the genome (Table 1). The distribution of sequence lengths (70–248,333 bp) was shown in Figure S1a, with an average length of 28,191.59 bp (Table 1). A total of 91.97% (Table 1) of the reads were mapped to the cattle genome assembly. Sniffles and PBSV identified 3665 and 29,285 SVs, respectively (Table 2). The identified SVs ranged from 32 bp to 2.62 Mb (mean size = 5676 bp) and 9 bp to 0.1 Mb (mean size = 592 bp) (Table S1), and their distribution across the whole genome was shown in Figure 2. The merging total number of SVs was 15,353 (1881 duplications and 13,472 deletions) (Table 2), covering 34 Mb of the whole genome (Table S1).

PacBio CLR sequencing yielded a total of 11,178,388 reads, representing 40-fold genome coverage, and they distributed in length between 53 and 369,285 bp (Figure S1b), with an average of 25,259.03 bp (Table 1). All reads were mapped to the cattle reference genome by pbmm2 (Table 1). Sniffles and PBSV identified 2578 and 1054 SVs, respectively (Table 2). The SV sizes identified by Sniffles ranged from 35 bp to 2.62 Mb, with an average size of 36,485 bp (Figure 2 and Table S1). For PBSV, the sizes ranged from 14 bp to 96 kb, and the mean size was 2377 bp (Figure 2 and Table S1). A total 2962 (1162 duplications and 1800 deletions) events covering 92 Mb (Table S1) of the whole genome were identified after merging (Table 2).

PacBio CCS sequencing generated 2,875,796 reads, representing 6× coverage of the genome. The distribution of sequence length (74–47,915 bp) is illustrated in Figure S1c, with an average length of 8763.78 bp (Table 1). All reads were mapped to the cattle reference genome by pbmm2 (Table 1). Sniffles and PBSV identified 289 and 29,922 putative SVs, respectively (Table 2). The SV sizes identified by Sniffles ranged from 34 bp to 3.6 Mb and had a mean size of 72,166 bp (Figure 2, Table S1). For PBSV, the sizes ranged from 8 bp to 100 kb, and the mean size was 722 bp (Figure 2 and Table S1). The total merging number of SVs was 19,492 (3891 duplications and 15,601 deletions) (Table 2), covering 41 Mb of the whole genome (Table S1).

### 3.2. SV Overlap

**Figure 3:**
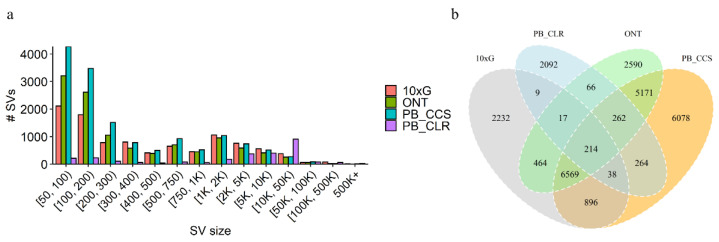
(**a**) Size distribution for SVs inferred from either long reads or Illumina/10 × G short reads. (**b**) Comparison between the four SV datasets.

In general, the total amount of SVs derived from short reads is much smaller than for the long-read-based inferences (Table 2). Most of the SVs were located between 50 bp to 200 bp, but long-read-based inferences can detect more large SVs (Figure 3a). Overall, these results show that across SVs accounts and sizes, long-read-based SV inference outperforms that of short reads. Between 45% and 60% of variants were called in at least one of the long-read data types, both of which were supported (Figure 3b). SVs called using PacBio CCS and ONT data showed high concordance (Figure 3b). The highest overlap with the results obtained from the short-read data was the PacBio CCS.

## 4. Discussion

The long reads generated by the third-generation sequencing technology can span tens of thousands of base pairs, which are tremendously serviceable in filling gaps in current references [28,29] and for the assembly of complicated genomic regions [29,30]. Meanwhile, they can also be helpful for the identification of large SVs. In this study, we presented a comparison of four sequencing datasets from the same cattle lung DNA sample. We sequenced the genome with Illumina/10 × G, ONT, and PacBio (CLR and CCS) sequencing technologies and subsequently analyzed for structural variations. We observed comparisons between various SV methods and how SV results differ for different sequencing technologies.

We identified a total of 21,164 SVs, which amount to 186 Mb covering 7.07% of the whole genome (Table 2). In general, except for PB CLRs, the number of SVs inferred from long-read-based inferences was greater than that of short-reads (Table 2). The CLR detected the least number of SVs, probably due to insufficient coverage, but it identified the most of large SVs and covered the most genomes (Figure 3a). When using 10× linked reads, we obtained 10,439 SVs, but there were 8207 SVs shared between short- and long-read technologies (Figure 3a). We showed that SVs called with PacBio CCS versus ONT data show high concordance, with more than 90% of SVs called with one platform also being called with the other (Figure 3a), which is consistent with human results [22]. Our results indicated a concordance between SVs inferred with ONT and PacBio CCS.

With the advancement of long-read sequencing, the higher quality of the reference assembly could further benefit the identification of SVs. Leonard et al. showed that 20× for HiFi or 60× for ONT sequencing was sufficient to produce two haplotype-resolved assemblies while retaining over 90% accuracy in detecting SVs when integrated into pangenomes [31]. With a combination of PacBio HiFi, Hi-C, and ONT ultra-long read sequencing, we could soon routinely obtain a Telomere-to-Telomere (T2T) assembly for livestock, as recently demonstrated for humans [32].

## 5. Conclusions

In this study, we generated four sequencing datasets and compared the SV results based on them. For each dataset, we identified SVs using two programs. Our results indicated a concordance between SVs inferred with ONT and PacBio CCS. The one with the most overlap with the results obtained by short-read data is PB CCS. Together, we found that long reads performed better than short reads in terms of SV detections.

## Figures and Tables

**Table 1:** Yield and alignment coverage statistics for the cattle lung sample across various sequencing platforms.

Platform	10 × G	PromethION	PacBio CLR	PacBio CCS
Number of reads	1,577,259,728	1,618,623	11,178,388	2,875,796
Mapped reads	1,532,221,733	1,488,641	11,178,388	2,875,796
Mapping rate (%)	97.14	91.97	100	100
Depth	55×	11×	40×	6×
Read min length	19	70	53	74
Read max length	150	248,333	369,285	47,915
Read mean length	133.94	28,191.59	25,259.03	8763.78

**Table 2:** Statistics over SVs identified by various methods.

Platform	Method	DEL	DUP	Total
10 × G	LongRanger	8242	73	8315
	LinkedSV	6415	38	6453
	Merge	10,325	114	10,439
ONT	PBSV	26,397	2888	29,285
	Sniffles	3497	168	3665
	Merge	13,472	1881	15,353
PB_CLR	PBSV	885	169	1054
	Sniffles	1340	1238	2578
	Merge	1800	1162	2962
PB_CCS	PBSV	23,353	6569	29,922
	Sniffles	190	99	289
	Merge	15,601	3891	19,492
Merge	SURVIVOR	16,289	4875	21,164

## Data Availability

The SV calls reported in this article are available in the Supplemental Material, and sequencing data are available upon request for research purposes.

## Supplementary Materials

The following supporting information can be downloaded at: https://www.mdpi.com/article/10.3390/genes13050828/s1. Figure S1: Length distribution for reads from ONT and PacBio sequencing runs. Table S1. Summary of identified SVs using different methods.
