# Supplementary figures and images for: Initial Analysis of Structural Variation Detections in Cattle Using Long-Read Sequencing Methods
Source: Genes (Basel). 2022 May 6;13(5):828. doi: 10.3390/genes13050828 (PMC9142105; doi:10.3390/genes13050828)

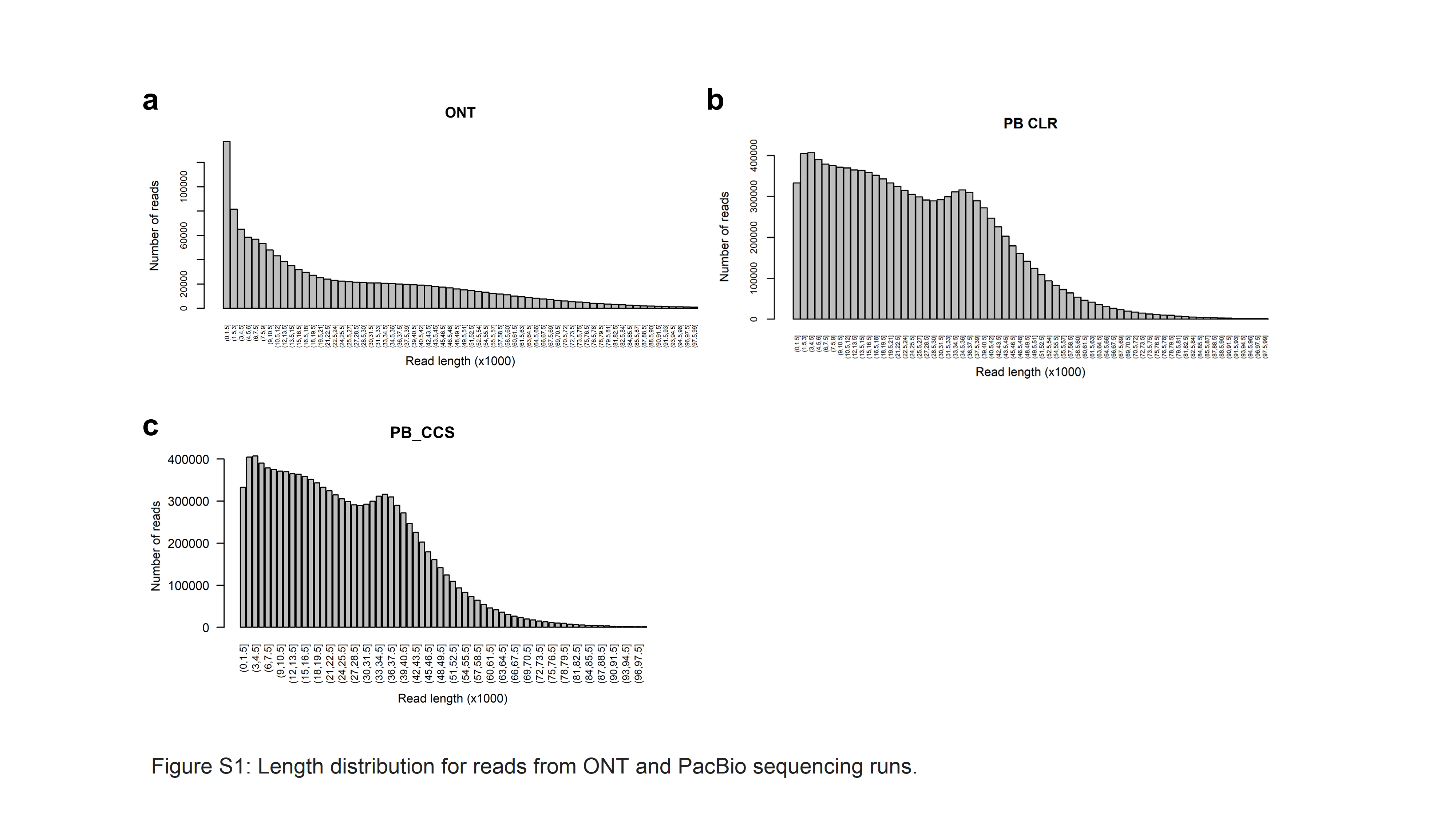

Supplement: Supplementary file 1 [file genes-13-00828-s001.zip › genes-1707366/Figure S1.png]
